# Impact of climate variability on exercise-induced bronchospasm in adolescents living in a semi-arid region

**DOI:** 10.31744/einstein_journal/2021AO5744

**Published:** 2021-09-17

**Authors:** Edinely Michely de Alencar Nelo, Jânio Luiz Correia, Hamilton Felipe Andrade Santos, José Pereira de Lima, Jéssica Thayani Santos Brandão, José Fernando Vila Nova de Moraes, Marco Aurélio de Valois Correia, Ricardo de Freitas-Dias

**Affiliations:** 1 Universidade de Pernambuco PetrolinaPE Brazil Universidade de Pernambuco, Petrolina, PE, Brazil.; 2 Universidade Federal do Vale do São Francisco PetrolinaPE Brazil Universidade Federal do Vale do São Francisco, Petrolina, PE, Brazil.; 3 Universidade de Pernambuco Programa de Pós-Graduação em Hebiatria CamaragibePE Brazil Programa de Pós-Graduação em Hebiatria, Universidade de Pernambuco, Camaragibe, PE, Brazil.

**Keywords:** Asthma exercise-induced, Humidity, Climate, Exercise, Adolescent

## Abstract

**Objective:**

To examine the impact of climate variability on the occurrence of exercise-induced bronchospasm in the rainy and dry seasons of a Brazilian semi-arid region.

**Methods:**

This sample comprised 82 adolescents aged 15 to 18 years, who were submitted to exercise-induced bronchospasm assessment on a treadmill and outdoors, during the rainy and the dry season. Anthropometric variables, sexual maturity and forced expiratory volume in the first second were analyzed. Air temperature and humidity, decline in forced expiratory volume in the first second (%) and frequency of bronchospasm were compared between seasons using the independent Student’s *t* test, the Wilcoxon and McNemar tests, respectively. The level of significance was set at p<0.05.

**Results:**

The mean age was 15.65±0.82 years. Air temperature, air humidity and decline in forced expiratory volume in the first second (%) differed between seasons, with higher air temperature and humidity in the rainy season (29.6ºC±0.1 and 70.8%±0.6 *versus* 28.5ºC±0.2 and 48.5%±0.6; p<0.05). The decline in forced expiratory volume in the first second (%) was greater in the dry season (9.43%±9.97 *versus* 12.94%±15.65; p<0.05). The frequency of bronchospasm did not differ between seasons.

**Conclusion:**

The dry season had a negative impact on forced expiratory volume in the first second in adolescents, with greater decrease detected during this period. Findings of this study suggested bronchospasm tends to be more severe under low humidity conditions.

## INTRODUCTION

Exercise-induced bronchospasm (EIB) is a transient bronchial constriction that happens after exercise, leading to a 10% or greater decrease in forced expiratory volume in the first second (VEF_1_) relative to baseline.^(^^1^^)^ The triggering mechanism includes some factors, such as individual susceptibility,^(^^2^^)^ exercise duration, and intensity and environment conditions, especially relative air humidity.^(^^3^^)^

The pathophysiology of EIB is directly related to exercise-induced hyperventilation and resultant low airway dehydration.^(^^4^^)^ This process affects the osmotic gradient in the epithelium, and stimulates the release of mediators involved in bronchospasm by nerves, epithelial and inflammatory cells.^(^^4^^)^

Apart from this pathophysiological process, climates with low relative air humidity levels (<50%) are associated with higher rates of bronchospasm.^(^^3^^)^ Hence, EIB tends to be more common in cold and dry ^(^^4^^,^^5^^)^ than in warm and humid climates.^(^^3^^,^^4^^)^

Most studies investigating EIB are conducted indoors (*i.e*., in controlled environments).^(^^1^^–^^4^^)^ However, external environmental factors may affect to occurrence of EIB.^(^^1^^–^^3^^)^ This study set out to elucidate the relation between climate variables (air temperature and relative humidity) and EIB, given the potential exposure of susceptible individuals to environmental conditions.^(^^3^^,^^6^^)^

## OBJECTIVE

To examine the impact of climate variability (air temperature and relative humidity) on exercise-induced bronchospasm development in adolescents, in the dry and the wet season of a Brazilian semi-arid region.

## METHODS

### Participants

This convenience sample comprised adolescents with no respiratory symptoms, aged 15 to 18 years,^(^^7^^)^ and living in a semi-arid region of Brazil. Adolescents with a history of respiratory infections in the 4 weeks before testing were excluded. Other exclusion criteria were pregnancy and self-reported cardiovascular, musculoskeletal or metabolic disorders. This study was conducted from March to April, and from August to November 2018, in the city of Petrolina (PE). Petrolina is located in the Brazilian Northeast and has a semi-arid climate (BSwh classification), with high temperatures (>22°C) and low rainfall (<250mm) in winter.^(^^8^^)^ The assent and informed consent forms were signed by adolescents or their guardians. This study was approved by the Research Ethics Committee of *Universidade de Pernambuco* (UPE), Brazil (CAAE: 81537517.2.0000.5207, opinion no. 2.701.140).

### Sample size calculation

Sample size was estimated by effect size calculation based on the primary endpoint and the percentage of EIB among adolescent students.^(^^4^^)^ *Post-hoc* analysis was conducted as follows: effect size of 0.2, α error <0.05, and β error <0.95. The estimated sample size corresponded to 82 volunteers. Procedures were performed using WinPepi software, version 11.65 for Windows. Sample size was large enough to detect significant differences between experimental periods for the variables air temperature and relative humidity (effect size larger than r=0.50; large effect), and VEF_1_ decline (%) (effect size larger than r=0.10, small effect).

### Study design

**Figure 1:**
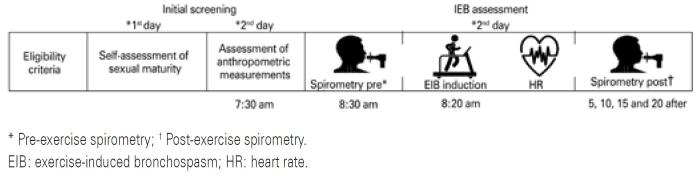
Experimental design

Volunteers were submitted first to self-assessment of sexual maturity,^(^^9^^)^ then to anthropometric measurements, spirometry, and the EIB testing. Tests were carried out during the rainy (March and April) and dry (August to November) seasons of the year 2018. Seasons were defined according to climate data recorded at Bebedouro agrometeorological weather station and provided by *Instituto Nacional de Meteorologia* (INMET).^(^^8^^,^^10^^)^ Air temperature and relative humidity were monitored during both experimental periods (Figure 1).

### Instruments and data collection

#### Sexual maturity

The sexual maturity status of male and female adolescents in this sample was determined using the Tanner Pubertal Development Scale, in compliance with self-assessment guidelines.^(^^9^^)^

#### Anthropometric data

Anthropometric variables were measured by a single examiner, in compliance with the International Society for the Advancement of Kinanthropometry (ISAK) guidelines.^(^^11^^)^ Total body mass and height were measured using a digital scale and stadiometer (W-200, Welmy, Brazil). Waist circumference was measured using a measuring tape (WCS, Mabbis, Brazil).^(^^12^^,^^13^^)^ The body mass index (BMI) was determined using the Quetelet index equation: BMI = weight (kg) / (height (m))². This index was used for body fat categorization into percentiles (below the 5^th^ percentile, underweight; 5^th^ to 84^th^ percentile, normal weight; 85^th^ to 94^th^ percentile, overweight; 95^th^ percentile or over, obesity).^(^^14^^)^

#### Spirometry

Respiratory capacity was assessed using a portable spirometer (Sx 1000, KoKo, Longmont, CO, United States). A minimum of three spirometry maneuvers using a nose clip was performed. The highest forced vital capacity (FVC), FEV_1_, and Tiffeneau index values obtained were used in the analysis; reproducibility patterns were accounted for.^(^^15^^)^ Volunteers were duly instructed about test day recommendations and test details, in compliance with American Thoracic Society (ATS) and *Sociedade Brasileira de Pneumologia e Tisiologia* (SBPT) guidelines.^(^^16^^)^

#### Exercise-induced bronchospasm testing

Exercise-induced bronchospasm testing was carried out on a treadmill (Master Super ATL, Inbramed, Porto Alegre, RS, Brazil), according to official ATS standards.^(^^1^^)^ Volunteers were first submitted to a warm-up consisting of 1 minute of walking below 85% of maximum heart rate (HRmax). In the second minute, intensity was progressively increased in such a manner that adolescents reached an intensity higher than 85% of HRmax in the third minute. This intensity was then maintained for six minutes. Maximum heart rate was determined using the following equation: 208 - (age x 0.7).^(^^16^^)^ Exercise intensity was determined according to heart rate (HR), which was monitored every 30 seconds using a heart rate monitor (V800, Polar, Brazil).

After test completion, spirometry was repeated four times, and FEV_1_ measured at 5, 10, 15, and 20 minutes. The FEV_1_% relative to baseline (pre-test) was then calculated using the following equation: [(%FEV^1^ = baseline FEV_1_ – post-test FEV^1^ x 100 / baseline FEV_1_)]^(^^1^^)^ to determine EIB positivity, defined as FEV_1_ decrease equal to or higher than 10% relative to baseline.^(^^1^^,^^2^^)^ To detect the greater percentage of FEV_1_ decline across selected time points, the maximum FEV_1_ decline (QMFEV_1_) was calculated using the equation [(%QMFEV_1_ = baseline FEV_1_ – lowest post-test FEV_1_ X 100 / baseline FEV_1_)].^(^^2^^)^ Exercise-induced bronchospasm was diagnosed whenever FEV_1_ declined by 10% or more after exercise, according to widely adopted guidelines.^(^^1^^,^^2^^,^^17^^–^^19^^)^

### Environmental variables

Air temperature and relative humidity in the rainy and the dry season were recorded at the time of testing using a digital thermo-hygrometer (Incoterm, São Paulo, SP, Brazil). Air temperature and relative humidity data provided by INMET were also used.^(^^10^^)^

### Data analysis

Data were processed and analyzed using software SPSS, version 22.0 for Windows. Data were entered using double typing and checked. Data normality was investigated using the Kolmogorov-Smirnov test followed by descriptive and inferential analysis. Height, temperature, baseline FVC, and baseline FEV_1_ were normally distributed. Continuous variables were summarized as mean, standard deviation and 95% confidence interval.

Air temperature and relative humidity in the rainy and the dry season were compared using the independent Student’s *t* test. Forced expiratory volume in one second decline (%) and frequency of EIB were compared using the Wilcoxon and the McNemar tests, respectively. For normally distributed variables, Cohen d was used to estimate effect size, as follows: small effect (<0.20), moderate effect (0.20 to 0.50), large effect (0.50 to 1.0), and very large effect (>1.0).^(^^20^^)^ For non-normally distributed variables, effect size was determined using Pearson’s r, as follows: small effect (r=0.10), medium effect (r=0.30) and large effect (r=0.50). The level of significance was set at 5% (p<0.05).

## RESULTS

This sample comprised 82 adolescents, mean age of 15.65±0.82 years, 43 (52.4%) males and 39 (47.6%) females. As to sexual maturity, boys (41.9%) and girls (41%) were at stage T4 (pubescent) of the Tanner scale. Adolescents were categorized as normal weight based on anthropometric measurements and BMI percentiles (45.75±31.30) (Table 1).

**Table 1:** Anthropometric characteristics of adolescents

Variables	Mean± SD	95%CI
Weight, kg	59.57±10.61	57.24-61.90
Height, m	1.70±0.09	1.67-1.71
BMI, kg/m²	20.64±3.42	19.89-21.39
WC, cm	72.21±7.23	70.62-73.80

SD: standard deviation; 95%CI: 95% confidence interval; BMI: body mass index; WC: waist circumference.

Baseline (pre-exercise) spirometry measurements (FVC, FVC% of predicted, FEV_1_ and FEV_1_% of predicted) did not differ between the rainy and the dry season (Table 2).

**Table 2:** Baseline spirometry characteristics of male and female adolescents

Variables	Rainy	Dry	Rainy *versus* dry	p value	Cohen’s d
Baseline FVC, L	3.77±0.73	3.82±0.86	- 0.05 (-0.16-0.05)	0.299	0.06
FVC% predicted %	111.99±33.13	113.79±36.18	-1.80 (-5.05-1.45)	0.274	2.93
Baseline FEV_1_, L	3.42±0.68	3.43±0.78	- 0.01 (-0.12-0.09)	0.798	0.01
FEV_1_% predicted %	109.95±36.62	110.01±35.49	-0.06 (-3.91-3.79)	0.974	0.00

Results expressed as means±standard deviation and seasonal effects (difference between group means, adjusted for baseline values, with 95% confidence intervals).

p<0.05 – Dependent Student’s *t* test.

FVC: forced vital capacity; FEV_1_: forced expiratory volume in the first second; L: liters.

**Figure 2:**
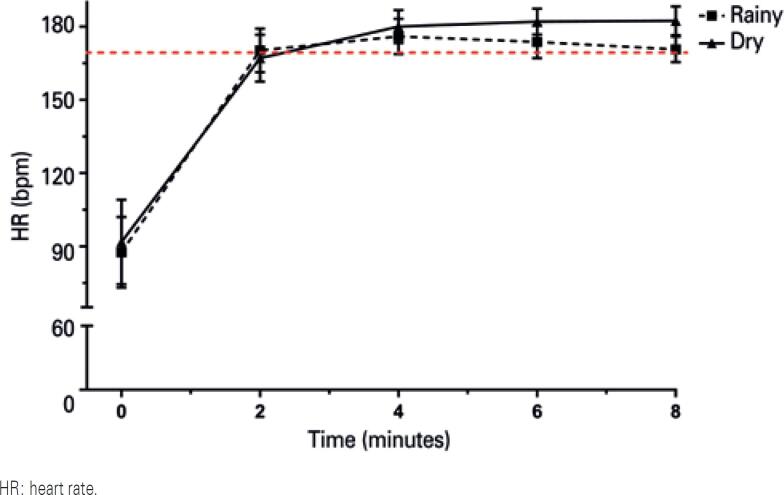
Exercise intensity variation during exercise-induced bronchospasm testing according to heart rate. The red dotted line represents the lower heart rate limit recommended for exercise intensities equivalent to 80% to 90% of maximum heart rate

The mean HR during EIB testing in the rainy and the dry season was 172.69±3.29bpm and 177.90±5.09bpm (87% and 90% of HRmax), respectively. Therefore, the recommended test intensity was achieved in both seasons (Figure 2).

**Figure 3:**
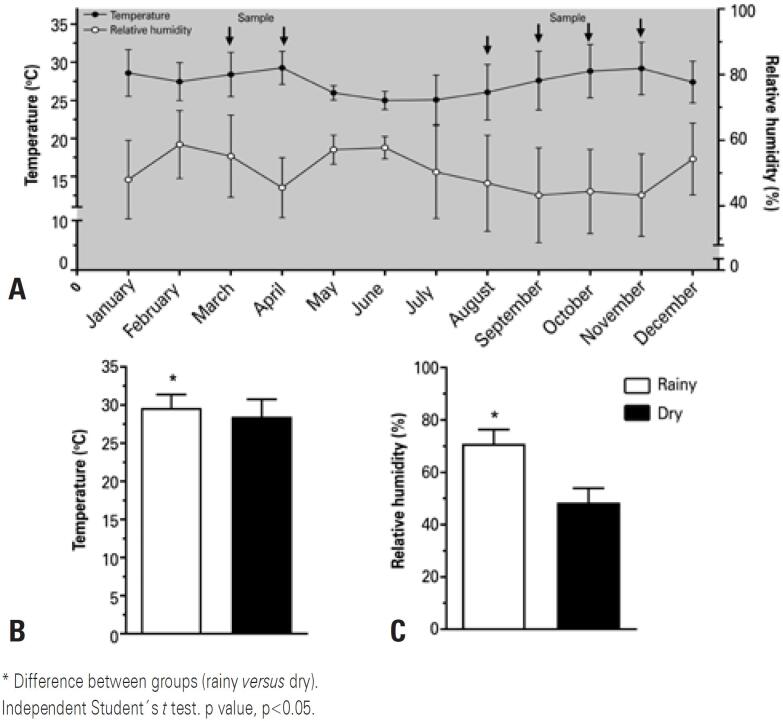
Intra-annual variability in air temperature and relative humidity in the year of 2017. A) Climate variability over the course of twelve months; B) Air temperature, comparisons between rainy and dry season; C) Relative humidity, comparisons between rainy and dry season

Air temperature and relative humidity recorded by INMET in selected months of the year ranged from 25.4°C to 33.4°C and 62% to 87% rainy season and from 23.8°C to 33.5°C and 37% to 72% dry season (Figure 3A). Comparative analysis of air temperature and relative humidity between the rainy and the dry season revealed differences (Figures 3B and 3C).

**Figure 4:**
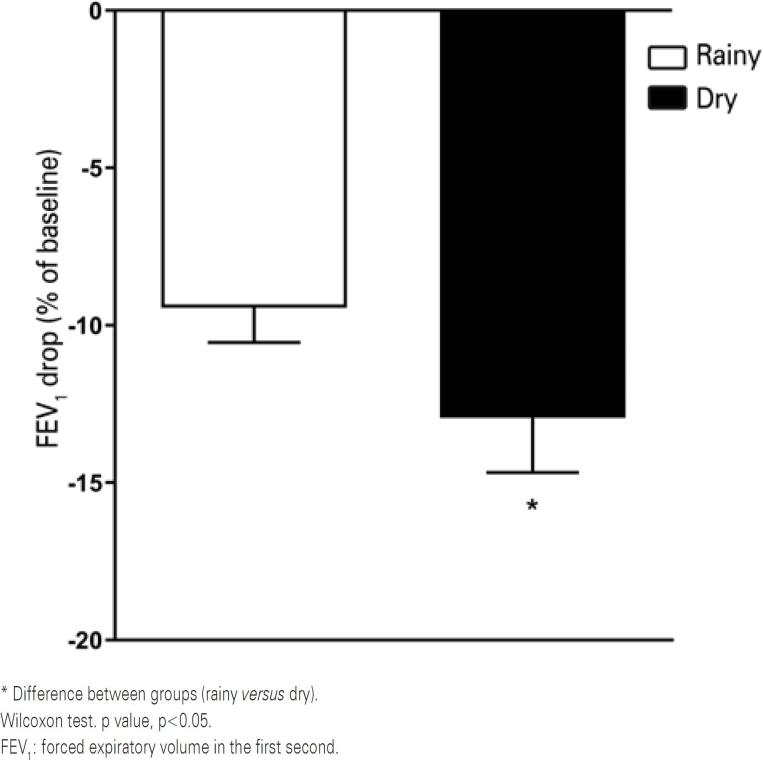
Comparison of forced expiratory volume decrease in the first second between seasons

As to EIB, the lowest post-test FEV_1_% predicted was 92.04%±29.45 and 96.70%±37.13 (rainy and dry season, respectively). The greatest decline in post-test FEV_1_ (%) relative to baseline differed between seasons, with greater percent decrease in the dry season and a small effect size (r=0.13) (Figure 4).

## DISCUSSION

This study examined the occurrence of EIB in adolescents living in a semi-arid region with a hot, dry climate, and low levels of relative air humidity throughout the year^(^^8^^,^^10^^)^ a factor associated with higher risk of EIB.^(^^3^^)^ The greater decline in FEV_1_ (%) occurred in the dry season, when air temperature and relative humidity were lower relative to the rainy season (in particular relative air humidity, with greater mean differences across seasons). According to current EIB assessment standards, the induction test in this study was effective, and climate variables (air temperature and relative humidity) were associated with FEV_1_ decline in adolescents in this sample. Findings of this study suggest EIB tends to be more severe in low relative air humidity conditions.

Methods of EIB identification and assessment are vital for accurate diagnosis. However, consensus regarding the ideal scenario for EIB testing^(^^6^^)^ and the gold standard for diagnosis in adolescents^(^^21^^)^ are lacking. To date, the only recommendation is the use of standardized induction tests. In this study, exercise testing was selected since it is thought to be an effective method to assess EIB.^(^^21^^)^

Exercise intensity control is an important aspect of exercise test standardization.^(^^1^^,^^3^^)^ In this study, the HR used to control test intensity reached the recommended zone of 80% to 90% of the predicted HRmax, or HR greater than 170bpm.^(^^1^^)^

In EIB (primary outcome) assessed according to FEV_1_, this spirometry variable is even more commonly used,^(^^6^^,^^22^^)^ and a percent decline in FEV_1_ ≥10% is thought to be sensitive enough for EIB assessment.^(^^1^^,^^23^^)^ Sensitivity may be enhanced in exercise testing conducted under low air temperature and relative humidity conditions,^(^^24^^)^ as in this study.

In a study examining climate variability and EIB, Johansson *et al.,*^(^^25^^)^ detected a similar percent decline in FEV_1_ in adolescents submitted to induction in dry air conditions (FEV_1_ decline, 18.7%±7.9). In a study by Park *et al*.,^(^^26^^)^ FEV_1_ decline was explained by a significant relation between air temperature and relative humidity (temperature: 9.9°C±1.2 *versus* 16.4°C±1.8; relative air humidity: 44.9%±1.4 *versus* 52.1%±2.4) and EIB positivity (61.4% *versus* 18.9%; p<0.05). In that study, EIB was more frequent in the cold, dry season.

Small temperature variation between experimental periods in this study may explain the smaller decline in FEV_1_. Although air temperature and relative humidity may impact EIB severity, this study revealed a greater decline in FEV_1_ in the dry season, when relative air humidity was lower, despite similar temperature ranges. High relative air humidity in the rainy season may also have prevented higher airway dehydration during exercise, since warm and humid environments are associated with lower risk of bronchial response.^(^^4^^)^

Literature data suggest relative air humidity plays a more significant role in bronchial responsiveness than air temperature.^(^^1^^–^^4^^)^ Dry climate is thought to induce more robust osmotic effects in response to more severe post-exercise airway dehydration in susceptible individuals.^(^^1^^,^^2^^,^^4^^)^ Such climate conditions have also been associated with higher incidence of EIB in individuals with no respiratory symptoms,^(^^1^^,^^2^^)^ given air conditioning (warming and humidification) capacity is lower during exercise due to enhanced ventilation.^(^^3^^,^^4^^)^

The impact of air temperature, and in particular of relative air humidity, on bronchial response is widely recognized. During high-intensity exercise, ventilation increases up to 30-fold relative to baseline, precluding appropriate conditioning of inhaled room air.^(^^1^^,^^2^^)^ This study was conducted in a semi-arid region, with warm, dry climate. Although cold, dry air is thought to trigger EIB. Rundell *et al.,*^(^^27^^)^ reported similar responses in room temperatures, and in cold, dry air conditions, suggesting inhaled air dryness is a more significant factor for EIB than air temperature.^(^^1^^,^^2^^,^^28^^)^ Furthermore, warm, humid environments may reduce or even prevent EIB development.^(^^29^^,^^30^^)^

According to ATS^(^^1^^,^^2^^)^ recommendations, bronchoprovocation tests should be conducted in environments with air temperature between 20°C to 25°C, and humidity below 50%. Important as these guidelines may be for test standardization purposes, they may preclude the assessment of individual or collective characteristics, which may be more appropriately investigated during activities undertaken in realistic environments. In this regard, this study makes significant contributions to outdoor assessment and simulates air temperature and humidity conditions experienced by adolescents in their routine activities. It also provides a seasonal assessment of post-exercise pulmonary function and explores typical air temperature and humidity conditions of the region.

Comparison of temperature variation between experimental periods limits potential comparison of findings with regions with low temperatures, in the rainy season, and is a limitation of this study. Future studies including factors associated with air quality (air pollutants) are warranted, given these factors may also impact EIB development.^(^^3^^)^

## CONCLUSION

Air temperature and relative humidity conditions in the dry season had a negative impact on forced expiratory volume in the first second in adolescents. The percent decline in forced expiratory volume in the first second was greater after physical exercise in the dry season of the semi-arid climate. Outdoor assessment contributed to the understanding of exercise-induced bronchospasm characteristics under climate conditions which more faithfully reflect those faced during games and playful activities, following bronchoprovocation tests conducted in a region with semi-arid climate.
