# Supplementary material for: Impact of climate variability on exercise-induced bronchospasm in adolescents living in a semi-arid region
Source: Einstein (Sao Paulo). 2021 Sep 17;19:eAO5744. doi: 10.31744/einstein_journal/2021AO5744 (PMC8448549; doi:10.31744/einstein_journal/2021AO5744)
Supplement: Translation

# Translation: Influência de alterações climáticas sobre a ocorrência do broncoespasmo induzido por exercício em adolescentes de uma região semiárida

**Authors:** Edinely Michely de Alencar Nelo, Jânio Luiz Correia, Hamilton Felipe Andrade Santos, José Pereira de Lima, Jéssica Thayani Santos Brandão, José Fernando Vila Nova de Moraes, Marco Aurélio de Valois Correia, Ricardo de Freitas-Dias

**Affiliations:** 1 PetrolinaPE Brasil Universidade de Pernambuco, Petrolina, PE, Brasil.; 2 PetrolinaPE Brasil Universidade Federal do Vale do São Francisco, Petrolina, PE, Brasil.; 3 CamaragibePE Brasil Programa de Pós-Graduação em Hebiatria, Universidade de Pernambuco, Camaragibe, PE, Brasil.

**Keywords:** Asma induzida por exercício, Umidade, Clima, Exercício físico, Adolescente

## Abstract

**Objetivo:**

Verificar a influência das alterações climáticas sobre o broncoespasmo induzido por exercício, nos períodos chuvoso e seco de uma região do semiárido brasileiro.

**Métodos:**

Foram submetidos à avaliação do broncoespasmo em esteira ergométrica, em ambiente externo, nos períodos chuvoso e seco, 82 adolescentes, com idades de 15 a 18 anos. Foram avaliadas as variáveis antropométricas, a maturação sexual e o volume expiratório forçado no primeiro segundo. Para comparação da temperatura e umidade, queda do volume expiratório forçado no primeiro segundo (%) e frequência do broncoespasmo entre os períodos, foram utilizados o teste *t* de Student independente, o teste de Wilcoxon e o teste de McNemar, respectivamente. O nível de significância adotado foi p<0,05.

**Resultados:**

A média de idade foi 15,65±0,82 anos. A temperatura, a umidade e a queda do volume expiratório forçado no primeiro segundo (%) diferiram entre os períodos, com valores de temperatura e umidade maiores no período chuvoso (29,6ºC±0,1 e 70,8%±0,6 *versus* 28,5ºC±0,2 e 48,4%±0,6; p<0,05). A queda do volume expiratório forçado no primeiro segundo (%) foi maior no período seco (9,43%±9,97 *versus* 12,94%±15,65; p<0,05), e não foi encontrada diferença da frequência do broncoespasmo entre os períodos.

**Conclusão:**

O período seco influenciou negativamente no volume expiratório forçado no primeiro segundo de adolescentes, observando maior percentual de queda dessa variável nesse período. De acordo com os achados, propõe-se uma maior gravidade do broncoespasmo induzido por exercício em condições de baixa umidade.

## INTRODUÇÃO

O broncoespasmo induzido por exercício (BIE) consiste em uma obstrução brônquica temporária após o exercício físico, caracterizada por redução maior ou igual a 10% do volume expiratório forçado no primeiro segundo (VEF_1_), comparado ao valor basal.^(^^1^^)^ Seu mecanismo de evocação compreende fatores relacionados à suscetibilidade individual,^(^^2^^)^ à intensidade e duração do exercício e a fatores ambientais, destacando-se entre estes a umidade relativa do ar.^(^^3^^)^

A fisiopatologia do BIE está diretamente ligada à hi­perventilação provocada pelo exercício físico, a qual gera a desidratação das vias aéreas inferiores.^(^^4^^)^ Esse processo ocasiona alteração do gradiente osmótico em relação ao epitélio, produzindo um estímulo para as células epiteliais, inflamatórias e fibras nervosas liberarem mediadores que podem estimular o broncoespasmo.^(^^4^^)^

Associado a esse processo fisiopatológico é reconhecido que, em condições climáticas com baixos valo­res percentuais de umidade relativa do ar (<50%), existe maior ocorrência de tal resposta brônquica.^(^^3^^)^ Diante disso, regiões com clima frio e seco apresentam risco maior de desencadeamento do BIE,^(^^4^^,^^5^^)^ enquanto em climas quente e úmido o risco é menor.^(^^3^^,^^4^^)^

Tendo em vista que grande parte dos estudos que avaliaram o BIE são realizados em ambientes internos (controlados)^(^^1^^–^^4^^)^ e as variações climáticas do ambiente externo podem influenciar na sua ocorrência,^(^^1^^–^^3^^)^ este estudo se faz necessário para esclarecer a relação entre as variáveis climáticas (temperatura e umidade relativa do ar) e o BIE, reconhecendo que indivíduos suscetíveis podem viver expostos a tais condições ambientais.^(^^3^^,^^6^^)^

## OBJETIVO

Verificar a influência das alterações climáticas (temperatura e umidade relativa do ar) na ocorrência do broncoespasmo induzido por exercício em adolescentes, nos períodos climáticos chuvosos e seco de uma região do semiárido brasileiro.

## MÉTODOS

### Participantes

A amostra, por conveniência, foi composta de adolescentes com idades de 15 a 18 anos,^(^^7^^)^ assintomáticos respiratórios, de uma região semiárida do território brasileiro. Foram excluídos aqueles que apresentaram qualquer infecção respiratória nas 4 semanas anteriores ao teste; gravidez; história de doença cardiovascular e musculoesquelética ou distúrbios metabólicos autorreferidos. A pesquisa foi realizada no período de março a abril e agosto a novembro do ano de 2018, na cidade de Petrolina (PE), situada no nordeste brasileiro, a qual apresenta clima do tipo BSwh, que corresponde a um clima semiárido, com temperaturas altas (>22°C) e chuvas escassas no inverno (<250mm).^(^^8^^)^ Os termos de assentimento e consentimento foram entregues aos adolescentes e assinados por eles e por seus responsáveis. Esta pesquisa foi aprovada pelo Comitê de Ética em Pesquisa da Universidade de Pernambuco (UPE), Brasil (CAAE: 81537517.2.0000.5207, parecer 2.701.140).

### Cálculo amostral

O tamanho da amostra foi estimado pelo cálculo do tamanho de efeito, considerando o desfecho principal e o percentual de BIE em adolescentes escolares.^(^^4^^)^ Foi realizada análise *post-hoc*, no qual o tamanho de efeito foi 0,2, o erro α <0,05 e o erro β <0,95, resultando no total de 82 voluntários. Para todos os procedimentos, foi utilizado o *software* WinPepi, versão 11.65 para Windows. O tamanho da amostra foi suficiente para detectar diferenças significativas entre períodos experimentais para as variáveis de temperatura e umidade relativa do ar, com tamanho de efeito maior que r=0,50 (efeito grande) e para a queda do VEF_1_(%), tamanho de efeito maior que r=0,10 (efeito pequeno).

### Delineamento do estudo

Inicialmente, todos os voluntários foram submetidos ao autoexame do estágio maturacional,^(^^9^^)^ seguido das mensurações das medidas antropométricas e da realização do teste de espirometria e do teste indutor do BIE. Todos os testes foram realizados nos períodos climáticos chuvoso (março e abril) e seco (agosto a novembro) do ano de 2018, sendo esses períodos definidos conforme os dados climáticos da estação agrometeorológica do Bebedouro, disponibilizados pelo Instituto Nacional de Meteorologia (INMET).^(^^8^^,^^10^^)^ Em ambos os períodos experimentais, a temperatura e a umidade relativa do ar foram monitoradas (Figura 1).

**Figura 1:** Desenho experimental

### Instrumentos e coleta de dados

#### Estágio maturacional

Para avaliar a o estágio maturacional dos adolescentes de ambos os sexos, foi utilizada a escala de autoclassificação maturacional do Estágio de *Tanner*, seguindo as recomendações para o autoexame individualizado.^(^^9^^)^

#### Antropometria

As variáveis antropométricas foram mensuradas por um único avaliador, seguindo as recomendações da *International Society for the Advancement of Kinanthropometry* (ISAK).^(^^11^^)^ A massa corporal total e a estatura foram medidas utilizando balança digital e estadiômetro (W-200, Welmy, Brasil). A circunferência da cintura foi mensurada utilizando uma fita métrica (modelo WCS, Mabbis, Brasil).^(^^12^^,^^13^^)^ O índice de massa corporal (IMC) foi determinado pela equação do índice de Quetelet em kg/m²: IMC (kg) = peso (kg) / (estatura (m))². Esse índice foi utilizado para classificação de gordura corporal por meio dos percentis (percentis abaixo do 5° para baixo peso, do percentil 5° ao 84° para a eutrofia, do percentil 85° ao 94° para sobrepeso e do percentil 95° em diante para obesidade).^(^^14^^)^

#### Teste espirométrico

Para a avaliação da capacidade respiratória, foi utilizado um espirômetro portátil (Modelo Sx 1000, KoKo, Longmont, CO, Estados Unidos), sendo realizadas, no mínimo, três manobras espirométricas, com uso de um clipe nasal. Para a análise dos parâmetros, foi escolhida a manobra que apresentou os maiores valores para as variáveis de capacidade vital forçada (CVF), VEF_1_ e índice de Tiffeneau, observando os padrões de reprodutibilidade.^(^^15^^)^ Os voluntários foram instruídos quanto às recomendações para o dia do teste, e o mesmo foi explicado previamente à realização, seguindo os procedimentos recomendados pela *American Thoracic So­ciety* (ATS) e pela Sociedade Brasileira de Pneumologia e Tisiologia (SBPT).^(^^16^^)^

#### Teste indutor do broncoespasmo induzido por exercício

O teste indutor do BIE foi realizado na esteira ergométrica (modelo Master Super ATL, Inbramed, Porto Alegre, RS, Brasil), seguindo a padronização oficial da ATS.^(^^1^^)^ Inicialmente, os voluntários realizaram um aquecimento, que consistiu em caminhada por 1 minuto em intensidade inferior a 85% da frequência cardíaca máxima (FCmáx). No segundo minuto, a intensidade foi aumentada gradualmente, até que, no terceiro minuto, alcançasse a intensidade superior a 85% FCmáx, a qual foi mantida por seis minutos. A FCmáx foi determinada pela equação: 208 - (idade x 0,7),^(^^16^^)^ e a intensidade do exercício foi avaliada com base na frequência cardíaca (FC), a qual foi monitorada a cada 30 segundos, por meio de cardiofrequencímetros (modelo V800, Polar, Brasil).

Ao término do teste indutor do BIE, foram realizados quatro novos testes espirométricos, nos quais a variá­vel VEF_1_ foi mensurada nos intervalos de tempo de 5, 10, 15 e 20 minutos. Em seguida, calculou-se o percentual do VEF_1_ em relação ao valor pré-teste, utilizando a equação [(%VEF_1_ = VEF_1_ pré-teste - VEF_1_ pós-teste x 100 / VEF_1_ pré-teste)]^(^^1^^)^ para detecção da positividade do BIE, definido como redução do VEF_1_ igual ou superior a 10% do valor basal (pré-teste).^(^^1^^,^^2^^)^ Também foi calculada a queda máxima de VEF_1_ (QMVEF_1_) por meio da equação [(%QMVEF_1_ = VEF_1_ pré-teste - menor VEF_1_ pós-teste X 100 / VEF_1_ pré-teste)],^(^^2^^)^ para detecção do maior percentual de redução do VEF_1_ nos tempos avaliados. Para a classificação do diagnóstico do BIE, adotou-se queda maior ou igual a 10% no VEF_1_ após exercício, conforme as recomen­dações das dire­trizes mundialmente aceitas.^(^^1^^,^^2^^,^^17^^–^^19^^)^

### Registo das variáveis ambientais

As variáveis climáticas de temperatura e umidade relativa do ar dos períodos chuvoso e seco foram registradas no momento dos testes, por meio de um termo-higrômetro digital (marca Incoterm, São Paulo, SP, Brasil). Também foram utilizados os dados de temperatura e umidade relativa do ar fornecidos pelo INMET.^(^^10^^)^

### Análises dos dados

Os dados foram processados e analisados no *software* SPSS, versão 22.0, para Windows, tendo sido inseridos por meio de digitação dupla com checagem posterior. Foi avaliada a normalidade, pelo teste de Kolmogorov- Smirnov, seguida de análise descritiva e inferencial. A estatura, a temperatura, a CVF pré e o VEF_1_ pré apresentaram normalidade dos dados. As variáveis contínuas foram resumidas em média, desvio-padrão e intervalo de confiança de 95%.

Para comparação das variáveis climáticas de temperatura e umidade relativa do ar entre os períodos chuvoso e seco foi realizado o teste *t* de Student independente; para a queda do VEF_1_(%), o teste de Wilcoxon e, para a frequência do BIE, o teste de McNemar. Adicionalmente, para estimar o tamanho de efeito, foi utilizado o d de Cohen para as variáveis com distribuição normal, com as seguintes classificações: efeito pequeno (<0,20), efeito moderado (0,20 a 0,50), efeito grande (0,50 a 1,0) e efeito muito grande (>1,0).^(^^20^^)^ Já para estimar o tamanho de efeito das variáveis de distribuição não normal, foi considerado o r de Pearson, com as classificações: efeito pequeno (r=0,10), efeito médio (r=0,30) e efeito grande (r=0,50). O nível de significância estabelecida foi de 5% (p<0,05).

## RESULTADOS

Participaram da pesquisa 82 adolescentes, com média de idade de 15,65±0,82 anos, sendo 43 (52,4%) do sexo masculino e 39 (47,6%) do sexo feminino. Referente à maturação sexual, tanto meninos (41,9%) quanto meninas (41%) encontravam-se ao estágio T4 (pubescente) da escala de Tanner. Os adolescentes foram classificados como eutróficos conforme os resultados antropométricos e a classificação do IMC por percentil (45,75±31,30) (Tabela 1).

**Tabela 1:** Características antropométricas de adolescentes

Variáveis	Média±DP	IC95%
Peso, kg	59,57±10,61	57,24-61,90
Estatura, m	1,70±0,09	1,67-1,71
IMC, kg/m²	20,64±3,42	19,89-21,39
CC, cm	72,21±7,23	70,62-73,80

DP: desvio-padrão; IC95%: intervalo de confiança de 95%; IMC: índice de massa corporal; CC: circunferência da cintura.

Sobre as variáveis espirométricas (CVF, CVF% do predito, VEF_1_ e VEF_1_% do predito), os valores basais (pré-exercício) foram semelhantes entre os períodos climáticos chuvoso e seco (Tabela 2).

**Tabela 2:** Características espirométricas basais de adolescentes de ambos os sexos

Variáveis	Chuvoso	Seco	Chuvoso *versus* seco	Valor de p	Cohen’s d
CVF pré, L	3,77±0,73	3,82±0,86	- 0,05 (-0,16-0,05)	0,299	0,06
CVF% predito, %	111,99±33,13	113,79±36,18	-1,80 (-5,05-1,45)	0,274	2,93
VEF_1_ pré, L	3,42±0,68	3,43±0,78	- 0,01 (-0,12-0,09)	0,798	0,01
VEF_1_% predito, %	109,95±36,62	110,01±35,49	-0,06 (-3,91-3,79)	0,974	0,00

Resultados expressos como médias±desvio-padrão e efeitos dos períodos climáticos (diferença de médias entre os grupos, ajustados para os valores basais, com intervalos de confiança de 95%).

p<0,05 – Teste *t* de Student dependente.

CVF: capacidade vital forçada; VEF_1_: volume expiratório forçado no primeiro segundo; L: litros.

A FC média alcançada no teste de indução do BIE foi de 172,69±3,29bpm no período chuvoso e de 177,90±5,09bpm no período seco, equivalentes a 87% e 90% FCmáx, respectivamente, o que confirma que a intensidade requerida no teste foi alcançada em ambos os períodos (Figura 2).

**Figura 2:** Variação da intensidade do exercício do teste de indução do broncoespasmo induzido por exercício, avaliada por meio da frequência cardíaca. A linha tracejada em vermelho representa o limite inferior da frequência cardíaca recomendada para o exercício em intensidade de 80% a 90% da frequência cardíaca máxima

A variação da temperatura e da umidade relativa do ar registrada pelo INMET durante os meses do ano foi de 25,4°C a 33,4°C e 62% a 87% no período chuvoso e de 23,8°C a 33,5°C e 37% a 72% no seco (Figura 3A). Quando se comparam a temperatura e a umidade relativa do ar entre os períodos chuvoso e seco dos dias do teste de indução, verificam-se diferenças entre as variáveis climáticas (temperatura e umidade relativa do ar) (Figura 3B e 3C).

**Figura 3:** Variação intra-anual de temperatura e umidade para o ano de 2017. A) Variações meteorológicas ao longo de doze meses; B) Comparação da temperatura durante o período chuvoso e seco do ano; C) Comparação da umidade relativa durante o período chuvoso e seco do ano

Referente ao BIE, o percentual do predito do menor VEF_1_ pós-teste de indução foi de 92,04%±29,45 no período chuvoso e 96,70%±37,13 no seco. Observou-se diferença quanto a maior queda do VEF_1_(%) em relação ao basal entre os períodos climáticos, sendo encontrado maior percentual de queda para o período seco com um tamanho de efeito pequeno (r=0,13) (Figura 4).

**Figura 4:** Comparação da queda do volume expiratório forçado no primeiro segundo entre os períodos climáticos

## DISCUSSÃO

Este estudo avaliou o BIE em adolescentes residentes em uma região de clima semiárido quente e seco, na qual há a peculiaridade climática regional de baixos percentuais de umidade relativa do ar durante o ano,^(^^8^^,^^10^^)^ sendo esse um fator relacionado a um maior risco de ocorrência do BIE.^(^^3^^)^ Observa-se maior percentual de queda do VEF_1_ no período seco, época do ano na qual a temperatura e a umidade relativa do ar foram menores em relação ao período chuvoso - em especial a umidade relativa do ar, que apresentou maior diferença de média entre os períodos. Conforme a presente padronização para a avaliação do BIE, foi possível considerar que o teste de indução utilizado foi efetivo e as variá­veis climáticas de temperatura e umidade relativa do ar influenciaram na queda do VEF_1_ nos adolescentes avaliados. Esta pesquisa proporciona o entendimento de uma maior gravidade do BIE em condições de baixa umidade relativa do ar.

Os métodos de identificação e avaliação do BIE são fundamentais para que se estabeleça um diagnóstico preciso, porém, ainda não existe consenso sobre o cenário ideal para a realização do teste de indução^(^^6^^)^ e nem sobre o padrão-ouro para o diagnóstico em adolescentes.^(^^21^^)^ Até o presente momento, é preconizado apenas que seja feita a padronização do teste de indução, tendo sido empregada, neste estudo, a indução do BIE por exercício, pelo fato de ser um método efetivo para avaliação do BIE.^(^^21^^)^

Um aspecto importante da padronização do teste de indução por exercício diz respeito ao controle da intensidade na qual o exercício é realizado.^(^^1^^,^^3^^)^ No presente estudo, a FC utilizada para o controle da intensidade no teste de indução alcançou o percentual recomendado de 80% a 90% da FCmáx prevista, que corresponde a uma FC superior a 170bpm.^(^^1^^)^

Tratando-se do desfecho primário do BIE, avaliado por meio do VEF_1_, essa variável espirométrica ainda é a mais utilizada,^(^^6^^,^^22^^)^ e o percentual de queda do VEF_1_ ≥10% é tido como suficiente para fornecer a sensibilidade necessária à avaliação do BIE.^(^^1^^,^^23^^)^ Tal sensibilidade pode ser aumentada quando usada à indução por exercício e em condições de baixa temperatura e umidade relativa do ar,^(^^24^^)^ conforme avaliado no presente estudo.

Analisando as variações climáticas e o BIE, Johansson et al.,^(^^25^^)^ encontraram resultados similares do percentual de queda do VEF_1_ em adolescentes submetidos à indução em ar seco (queda do VEF_1_ de 18,7%±7,9). Já em estudo de Park et al.,^(^^26^^)^ a queda do VEF_1_ foi explicada por uma relação significativa entre a temperatura e a umidade relativa do ar (temperatura: 9,9°C±1,2 *versus* 16,4°C±1,8, e umidade relativa do ar: 44,9%±1,4 *versus* 52,1%±2,4) e a positividade do BIE (61,4% *versus* 18,9%; p<0,05), sendo possível observar que a maior ocorrência do BIE foi encontrada no período em que o clima era mais frio e seco.

No presente estudo, a pequena variação de temperatura entre os períodos experimentais pode ter influenciado na menor queda do VEF_1_. Quanto à gravidade do BIE, embora seja observado que tanto a temperatura quanto a umidade relativa do ar podem ser fatores influenciadores, neste estudo foi encontrada maior queda do VEF_1_ no período seco, quando a umidade relativa do ar foi menor na mesma faixa de temperatura. Além disso, no período chuvoso, a umidade relativa do ar elevada pode ter prevenido maior desidratação das vias aéreas durante o exercício, visto também ser reconhecido que o ambiente quente e úmido está associado a menor risco de ocorrência da resposta brônquica.^(^^4^^)^

A literatura aponta a criticidade da umidade relativa do ar em detrimento da temperatura sobre o grau de responsividade brônquica,^(^^1^^–^^4^^)^ sendo reconhecido que o clima seco pode contribuir com maiores efeitos osmóticos, em resposta a maior desidratação da via aérea após o exercício em indivíduos mais suscetíveis.^(^^1^^,^^2^^,^^4^^)^ Também nessa condição climática, pode-se detectar maior incidência de BIE em assintomáticos respiratórios,^(^^1^^,^^2^^)^ pois, durante a prática do exercício, há redução da capaci­dade de condicionamento (aquecimento e umidifi­cação) do ar pelo aumento da ventilação.^(^^3^^,^^4^^)^

A influência da temperatura e, especialmente, da umidade relativa do ar é fator bem conhecido na resposta brônquica, uma vez que, durante o exercício intenso, ocorre aumento da ventilação de até 30 vezes os níveis basais, o que impede o condicionamento do ar inspirado do ambiente de forma adequada.^(^^1^^,^^2^^)^ O clima da região estudada é classificado como semiárido, quente e seco. Embora tenha sido sugerido que o ar frio e seco é um gatilho para o BIE, Rundell et al.,^(^^27^^)^ observaram respostas semelhantes em temperatura ambiente e em ar seco e frio, indicando que a secura do ar inalado, e não a temperatura, é o componente que mais contribui para tal resposta.^(^^1^^,^^2^^,^^28^^)^ Além disso, ambientes quentes e úmidos podem reduzir e praticamente bloquear o BIE.^(^^29^^,^^30^^)^

A ATS^(^^1^^,^^2^^)^ recomenda que o teste de broncoprovocação seja realizado em local com temperatura entre 20°C e 25°C e umidade inferior a 50%. Essas diretrizes são importantes para padronizar o exame, mas podem deixar de lado alguma característica individual ou coletiva, que seja melhor observada quando as atividades e brincadeiras do indivíduo sejam avaliadas o mais próximo da realidade possível. Nesse sentido, a atual pesquisa acrescenta conhecimento no que se refere à avaliação em campo aberto e simula as condições de temperatura e umidade às quais os adolescentes estão expostos em suas práticas corriqueiras, além de avaliar os efeitos da função pulmonar pós-exercício sazonalmente, explorando condições de temperatura e umidade bem característicos da região.

A limitação deste estudo é que a comparação da variação da temperatura entre os períodos experimentais limita as possíveis comparações dos nossos achados com outras regiões com condição climática de baixas temperaturas no período chuvoso. Também, sugere-se que estudos futuros incluam a análise de outros fatores relacionados à qualidade do ar (agentes poluentes), visto que também podem exercer influência na ocorrência do BIE.^(^^3^^)^

## CONCLUSÃO

As condições climáticas de temperatura e umidade relativa do ar do período seco influenciaram negativamente no volume expiratório forçado no primeiro segundo em adolescentes. Houve maior percentual de queda do volume expiratório forçado no primeiro segundo após a prática do exercício físico no período seco do clima semiárido. A avaliação em campo aberto possibilitou maior entendimento das características do broncoespasmo induzido por exercício em condições climáticas mais próximas da realidade em jogos e brincadeiras, após teste de broncoprovocação em uma região de clima semiárido.
